# Corrosion Study on Duplex Stainless Steel UNS S31803 Subjected to Solutions Containing Chloride Ions

**DOI:** 10.3390/ma17091974

**Published:** 2024-04-24

**Authors:** Lucas Menezes de Souza, Elaine Pereira, Thiago Barreto da Silva Amaral, Sergio Neves Monteiro, Afonso Rangel Garcez de Azevedo

**Affiliations:** 1 LAMAV—Advanced Materials Laboratory, UENF—State University of the Northern Rio de Janeiro, Av. Alberto Lamego, 2000, Campos dos Goytacazes 28013-602, RJ, Brazil; 202011220016@pq.uenf.br (L.M.d.S.); elainecp@pq.uenf.br (E.P.); 2 Corrosion Laboratory, IFES—Federal Institute of Education, Science and Technology of Espírito Santo, Av. Vitória, 1729, Jucutuquara, Vitória 29040-780, ES, Brazil; tbsamaral@gmail.com; 3 Materials Science Program, IME—Military Institute of Engineering, Praça Gen. Tibúrcio, 80, Rio de Janeiro 22290-270, RJ, Brazil; snevesmonteiro@gmail.com; 4 LECIV—Civil Engineering Laboratory, UENF—State University of the Northern Rio de Janeiro, Av. Alberto Lamego, 2000, Campos dos Goytacazes 28013-602, RJ, Brazil

**Keywords:** duplex stainless steel, pitting corrosion, uniform corrosion, hardness, tension

## Abstract

In the present work, the influence of a corrosive environment and temperature on the corrosion resistance properties of duplex stainless steel S31803 was evaluated. The corrosive process was carried out using solutions of 1.5% HCl (m/m) and 6% FeCl_3_ (m/m), at temperatures of 25 and 50 °C. The microstructure of UNS S31803 duplex stainless steel is composed of two phases, ferrite and austenite, oriented in the rolling direction, containing a ferrite percentage of 46.2% in the rolling direction and 56.1% in the normal direction. Samples, when subjected to corrosive media and temperature, tend to decrease their mechanical property values. It was observed, in both corrosive media, that with increasing test temperature, there is an increase in the corrosion rate, both uniform and pitting. The sample in HCl solution obtained a uniform corrosion rate of 0.85% at 25 °C and 0.92% at 50 °C and pitting rates of 0.77% and 1.47% at the same temperatures, respectively. When tested in FeCl_3_ solution, it obtained uniform corrosion of 0.0006% and 0.93% and pitting of 0.53% and 18.5%, at the same temperatures. A reduction in dissolution potentials is also noted, thus characterizing greater corrosion in the samples with increasing temperature.

## 1. Introduction

Stainless steels are iron-based alloys that contain a minimum of 11% Cr, an amount sufficient to provide a highly protective chromium oxide layer in unpolluted atmospheres. Other elements can also be added to this alloy in order to improve its characteristics, such as nickel, manganese, molybdenum, copper, titanium, silicon, niobium, aluminum, sulfur, and selenium. Its most used classification is based on the microstructure that they present at room temperature. In this condition, four groups are considered: (i) martensitic, (ii) ferritic, (iii) austenitic, and (iv) duplex. Duplex stainless steel was developed with the aim of associating high mechanical properties and resistance to corrosion, as the other three classes did not present an adequate combination [1,2,3].

Duplex stainless steels are part of a class of materials with a two-phase microstructure, continuous a ferritic matrix and islands of austenite, with approximately equal volume fractions (50 wt.%), thus combining characteristics of both alloys, such as, for example, immunity to stress corrosion, good ductility, good toughness, and good weldability. Intermetallic phases can precipitate at high temperatures and impair the properties of this material. A very harmful phase that can form is the sigma phase, a hard, fragile, and chromium-rich phase, which promotes the depletion of the matrix in relation to this element [1,2].

Duplex stainless steel S31803 has a yield strength of 450 MPa, higher than austenitic stainless steels that have this property around 300 MPa, due to its low percentage of nickel. Other alloying elements in smaller proportions can also be added. Molybdenum improves corrosion resistance and balances the microstructure of steel; nitrogen increases its mechanical strength and manganese increases nitrogen solubility in the material and partially replaces nickel [4,5].

The energy, chemical, petrochemical, nuclear, and oil and gas industries are increasingly employing new materials that combine high mechanical strength, corrosion resistance, and durability [6]. Therefore, the use of duplex stainless steel has been increasingly explored, due to its excellent combination of high corrosion resistance and high mechanical strength, combined with good toughness. S31803 duplex stainless steel is used in the production of flexible tubes for oil extraction and can also be applied to tanks for storing chemical products and equipment for the cellulose and paper segment [5,7].

Flexible pipelines are tubular structures, formed by the superimposition of metallic layers, with a structural function, and polymeric ones, with a waterproofing function, which give them the capacity to resist the pressure of the internal fluid and external hydrostatic pressure, due to the depth and dynamic loads of operation [5,7].

Corrosion can be defined in different ways, but in a universal aspect this term means the attack and deterioration of a metallic material, particularly by the action of the environment. This attack comes from a chemical or electrochemical action of the environment, combined or not with mechanical efforts [7,8].

The corrosion process of metallic alloys in environments in the presence of some liquid electrolyte, mainly aqueous media, is electrochemical in nature. Therefore, two or more electrochemical reactions occur, namely the loss and gain of electrons and the electrical and ionic conduction between cathode and anode. One of the reactions is the oxidation or anodic reaction, in which the loss of electrons occurs, generally in the metal or metallic alloy (Me) [9,10,11]. This reaction is rendered as follows:

Me → Me^n+^ + ne (1)

In this reaction, the metal increases its oxidation number (ne) and becomes a non-metallic state (Me^n+^ + ne). However, simultaneously, the reduction reaction or cathodic reaction takes place, in which electrons are gained. Some of the most common reactions found in the corrosion of metals in aqueous environments are presented in the following equations:

1. Hydrogen reduction (in a deaerated acid medium)

2H^+^(aq.) + 2e− → H_2_(g) (2)

2. Water reduction (in a neutral or basic deaerated medium)

2H_2_O(l) + 2e− → H_2_(g) +2OH^−^(aq.) (3)

3. Reduction of dissolved oxygen in an aerated acidic medium

O_2_(g) + 4H^+^(aq.) + 4e− → 2H_2_O(l) (4)

4. Reduction of dissolved oxygen in an aerated neutral or basic medium

O_2_(g) + 2H_2_O(l) + 4e− → 4OH^−^(aq.) (5)

For metal or alloy corrosion to occur, the anodic and cathodic reactions must occur simultaneously. Several cathodic reactions can occur during anodic dissolution, but electrochemical reactions will come into equilibrium whenever no external electrical source is acting. In other words, the reaction rate of reduction reactions is equal to that of oxidation. At this moment, each electrode will assume an equilibrium potential, also called corrosion potential. To study the corrosion phenomena and parameters of a metal or alloy under certain conditions, it is necessary to subject the system to external potentials or currents in order to evaluate the electrochemical response [9,10,11].

Due to corrosion being a spontaneous process, the deterioration caused in materials represents harmful and undesirable changes, such as wear, chemical variations, or structural modification and, in certain cases, making the material unsuitable for use. For the most diverse activities, such as chemical, petrochemical, oil, naval, and civil construction, among others, corrosion problems are frequent, causing the most diverse losses [11,12].

Corrosion resistance is the most important property of stainless steels, and it is the reason for their existence and continued use. Chromium at rates greater than 10%, when added to steel, achieves a rapid reduction in corrosion rates, due to the formation of a protective film. This film is an oxide that protects the steel from attack in aggressive environments. A chromium content of at least 11% is necessary to obtain a compact and continuous passive film. Passivity increases to a level of 17% chromium, and this is the main reason why many stainless steels contain 17 to 18% chromium [4,12].

The passive film on stainless steel is complex, composed of a mixture of metal oxides and hydroxides or oxyhydroxides, with the films possibly containing bound water. Cr and Ni oxides can be seen, as well as Fe. In the inner region, Cr^3+^ and Ni^2+^ ions are present, while the outer region of the film contains only the Cr^3+^ ion. When stainless steels have molybdenum as an alloying element in their composition, generally the Mo^4+^ ion is incorporated into the internal region of the film, while the Mo^6+^ ion is present in the external layer. Molybdenum ions act as a kind of selective cations, forming a bipolar passive film on the outer layer, with the inner layer containing Cr_2_O_3_, being anion-selective. In this way, the passage of active anions into the film and the passage out of cations produced by metallic dissolution at the metal/oxide interface is not favored [4,12].

Although the duplex stainless steel UNS S31803 has already been studied by many researchers [5,9,13,14], these studies are more focused on the material when subjected to different types of processes, such as the study of its corrosion resistance in friction-welded joints or for weld evaluation of the corrosive process after tempering heat treatment, among others. However, no work was found covering all the parameters detailed in this research, whose main objective was to evaluate the behavior of UNS S31803 steel in the as-received state and after being subjected to corrosive media in conditions close that of flexible pipes used in oil platforms, therefore being of great practical importance.

It is worth mentioning that the microstructural characterization was performed using optical and confocal microscopy techniques. The determination of the mechanical properties of hardness and tension, the uniform and pitting corrosion rate, and the evaluation of the polarization measurements of duplex stainless steel S31803 after being subjected to the corrosion process in two different media at RT was carried out at a temperature of 50 °C. The corrosive solutions used were 1.5% (m/m) hydrochloric acid (HCl) and 6% (m/m) ferric chloride (FeCl_3_), with the aim of evaluating corrosion in the presence of chlorides. The concentration of the HCl solution was dimensioned according to the research carried out by Gunn et al., [2] and the exposure time according to the ASTM G31 [15] standard, while the concentration and exposure time of the FeCl_3_ solution were established from the ASTM G48 standard [16].

## 2. Materials and Methods

### 2.1. Material

The material used in this work was duplex stainless steel S31803 supplied by TechnipFMC Brazil (Rio de Janeiro, Brazil) and received in the form of sheets that were 300 mm long, 100 mm wide, and 25 mm thick. It is important to point out that the specimens used for carrying out the tests were sectioned in the lamination direction of the material.

### 2.2. Metallographic Characterization

The specimens were metallographically prepared in a conventional manner with cutting, embedding, sanding, polishing, and etching. The chemical attack used was the Beharra II solution provided by Synth (São Paulo, Brazil), with 0.3 g of potassium metabisulphite, 20 mL of hydrochloric acid, and 100 mL of distilled water, using the immersion technique for 10 s.

The metallographic characterization of the sample in the as-received state was performed in the directions of lamination and normal to the lamination of the plate, using the Olympus Ols 4000 confocal microscope (supplier Olympus, Sao Paulo, Brazil). Image contrast and Stream Essentials 2.4 software were used to determine the quantitative analysis of the phases present. The evaluation of the specimens after the corrosion tests was also performed by confocal microscopy (supplier Olympus Brazil).

### 2.3. Corrosion Test

For the corrosion test, twenty-four specimens were used, twelve of which were machined according to ABNT NBR ISO6892 [17] for tensile testing, while the others were manufactured according to NACE RP 0775 [18] in the form of mass loss coupons, with dimensions of 50 × 25 mm. They were tested in two different corrosive media, namely hydrochloric acid and ferric chloride.

The hydrochloric acid solution (supplier Merck, Cajamar, Brazil) was used at a concentration of 1.5% (m/m); the tests were carried out at temperatures of 25 and 50 °C, according to the research carried out by Gunn et al., [2], lasting for 167 h, according to ASTM G31 [15]. The test with the ferric chloride solution (supplier Merck Brazil) was carried out according to ASTM G48 method A [16]. The solution concentration was 6% (m/m), at temperatures of 25 and 50 °C, and the test lasted 72 h.

### 2.4. Mechanical Properties

Hardness values were determined using an automatic Tukon 2500 Durometer, Wilson Hardness brand (Norwood, MA, USA), using the Vickers method, in accordance with ABNT NBR ISO 6507 [19]. The material was tested with a load of 10 HV for 10 s.

The tensile tests were carried out at RT in a universal testing machine, model DL6000 supplier EMIC, with an extensometer attached, at a speed of 2 mm/min. Three specimens were used per test parameter, and these were machined according to the ABNT NBR ISO 6892 standard [17]. The determined mechanical properties were yield stress (MPa), tensile strength limit (MPa), and ductility (%EL and %RA). The ductility %EL and %RA are just two different methods of calculating ductility, where the first is based on the elongation of the material and the second is based on the reduction in the area in the necking region.

### 2.5. Determination of Uniform and Pitting Corrosion Rate

The uniform and pitting corrosion rates were determined following the guidelines of the NACE RP 0775 [18] standard. It is noteworthy that the uniform corrosion rate was determined by the mass loss technique, using a scale model AX200 SHIMADZU, Tokyo, Japan, with a precision of four decimal digits, while the pitting rate took into account the deepest pit developed in the material structure according to the exposure time in the corrosive medium. Both rates were determined in mm/year according to the Equations (1) (uniform) and (2) (pitting), provided as follows:

(6) $$CR=\frac{W*365*1000}{\begin{array}{c} ATD \end{array}}$$

where:

CR = average corrosion rate, millimeters per year (mm /year);

W = mass loss, grams (g);

A = initial exposed area of the coupon, square (millimeters (mm^2^);

T = exposure time, days (d);

D = coupon metal density, grams per cubic centimeter (g/cm^3^).

(7) $$PR(mm/ano)=\frac{Depth\;of\;deepest\;pit\left(mm\right)*365}{exposuretime(days)}$$

### 2.6. Polarization Test

The electrochemical polarization tests were carried out on samples with dimensions of 20 × 10 mm, at temperatures of 25 and 50 °C, using corrosive media with solutions of HCl 1.5% (m/m) and FeCl_3_ 6% (m/m). The Autolab potentiostat (supplier Metrohm, São Paulo, Brazil) was used to survey the polarization curves. The system includes the following: (i) a thermometer to check the temperature of the solution, where the sample is immersed, (ii) the calomel reference electrode (E = +0.242 V), and (iii) the platinum counter electrode.

The potentiodynamic test started after 1800 s of the sample being immersed, for stabilization and determination of the open circuit potential, followed by a sweep of 1.0 mV/s in the sample, in a range of −0.8 to 2.0 V, with a minimum current of 10 nA and a maximum amplitude of 1 A. It is noteworthy that two potentiodynamic tests were carried out for each specimen in order to evaluate the reproducibility of the tests.

## 3. Results

### 3.1. Microstructural Characterization in the As Received State

**Figure 1:**
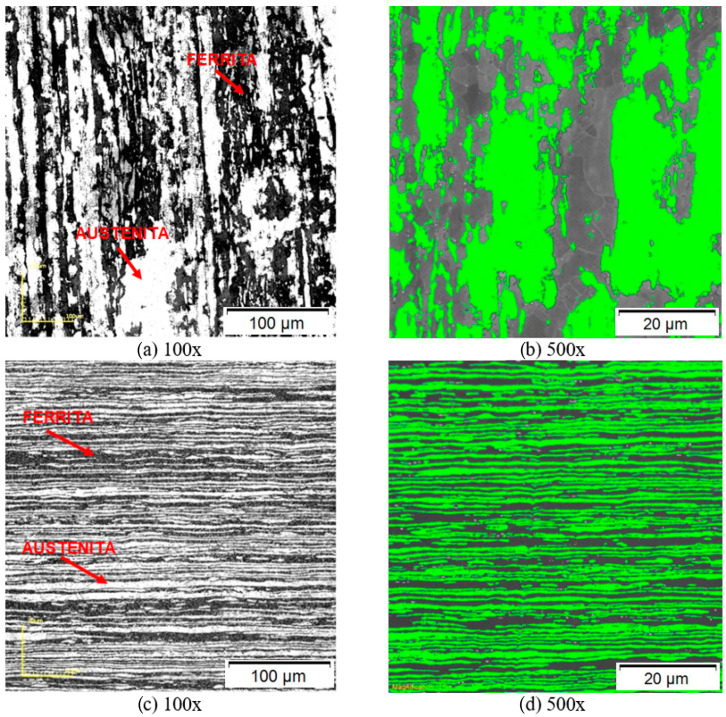
Microstructural characterization of S31803 duplex stainless steel in the as-received state using confocal microscopy (**a**,**c**) and image contrast (**b**,**d**), in the lamination direction (**a**,**b**) and normal (**c**,**d**).

The microstructure of duplex stainless steel S31803, in the as-received state, shows an elongated morphology in the rolling direction, consisting of alternating lamellae of the austenitic (light layer) and ferritic (dark layer) phases. The lamellar microstructure is formed because the α-γ interface energy is thermodynamically lower, when compared to the energies of the α-α and γ-γ boundaries [20]. Figure 1 presents the microstructure of the steel in the as-received staet in the rolling direction (a,b) and normal to the sheet rolling direction (c,d). The morphological aspect is already well established and corroborates the literature [21,22,23].

Many duplex stainless-steel applications [14,24,25] require the material to be rolled prior to use. In most cases this material shows a typical microstructure with elongated ferrite grains isolated from each other by other austenite grains. In particular, the ferritic phase is irregular in length, in cross-sectional area and in the distance that they separate. In addition, they are aligned mainly along the rolling direction. The rolling process further leads to the development of texture, elongated grains/stripes, and directional order [2,25,26,27].

Ferrite presents thinner lamellae than austenite after high strains, because the smaller number of sliding systems and the low stacking energy promote a higher hardening rate of the austenitic phase. Therefore, the deformation is more concentrated in the ferritic phase [2,21,22].

The quantitative analysis of the phases, determined by the contrast of the images, as in Figure 1b,d is presented in Table 1. Note that the duplex stainless steel S31803 has an approximate balance of 50 vol% of the austenite and ferrite fractions, in both senses evaluated. According to the literature [4,24,28], the percentage of austenite and ferrite phases present in the microstructure of duplex stainless steels must be balanced and may have a minimum fraction of up to 40 vol% of austenite.

### 3.2. Characterization of Corrosion after Tests

**Figure 2:**
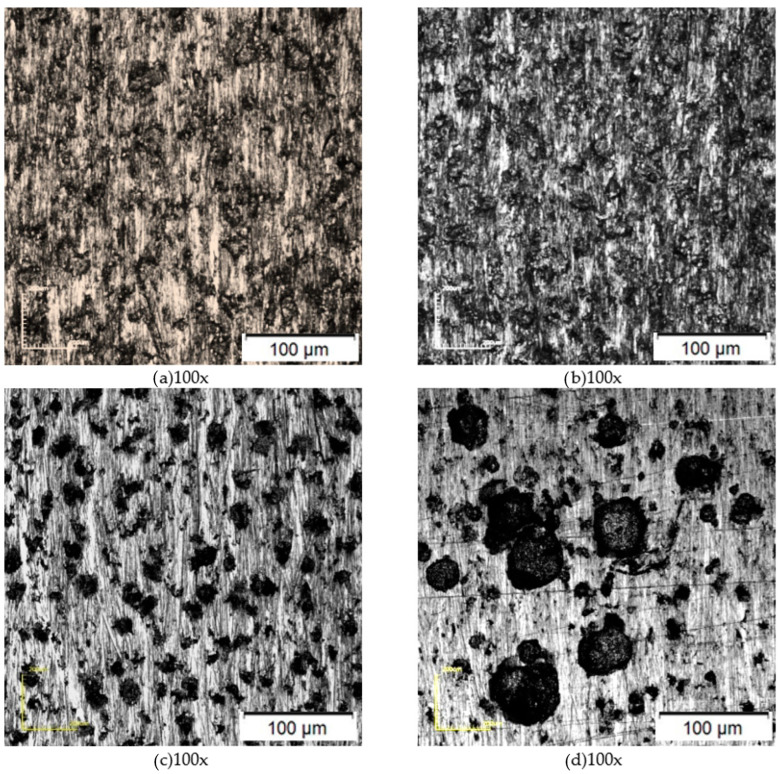
Characterization by confocal microscopy after corrosion tests in hydrochloric acid (**a**,**b**) and ferric chloride (**c**,**d**) at temperatures of 25 °C (**a**,**c**) and 50 °C (**b**,**d**).

The characterization of the specimens after the corrosion test in hydrochloric acid (HCl) and ferric chloride (FeCl_3_) media, at temperatures of 25 and 50 °C, is shown in Figure 2. In all microstructures, it was possible to observe the rough aspect of the uniform corrosion and corrosion pits, evidenced by small cavities in the metal surface. In the samples subjected to a temperature of 50 °C, the pits could be seen even with the naked eye. Pitting corrosion is more evident in the samples subjected to the FeCl_3_ solution, as in Figure 2c,d. This solution is normative, ASTM G48 [16], and widely used in laboratory corrosive tests, as it is highly aggressive, accelerating the corrosive process. With the use of the FeCl_3_ solution, a high anodic overvoltage is chemically applied to the material, through the Fe^3+^/Fe^2+^ reduction–oxidation pair, which has a high potential of approximately +0.45 V (ECS), ensuring that the pit is overcome, particularly in this chloride-heated medium. The low pH of the medium ensures that the solution inhibits the repassivation process and reduces the passive film stability. It is well known in the literature [2,20] that pitting corrosion is evident in stainless steels when subjected to a solution containing chloride ions. Chloride ions, being strong acid anions, are small in size and exhibit high diffusivity, and they are responsible for developing punctual defects in passive films, leading to punctate corrosion [2,28].

Regarding the increase in temperature in corrosive media, an increase in the area and depth of the pits was observed when subjected to the same solutions at temperatures of 25 and 50 °C, as in Figure 2a,d. This behavior becomes more evident in the corrosive environment of FeCl_3_, as in Figure 2d. The literature indicates [28,29] that the increase in the temperature of the corrosive environment promotes a decrease in protection, providing greater susceptibility to corrosion. The increase in temperature is capable of accelerating the process due to the increase in electrolyte conductivity, diffusion, and ionic solubility, which is in agreement with what was observed.

### 3.3. Mechanical Properties

**Figure 3:**
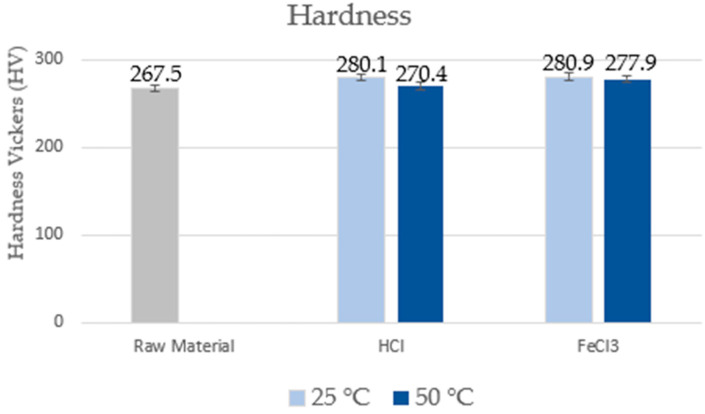
Average Vickers hardness values.

The Vickers hardness values for duplex stainless steel S31803 in the as-received state and after corrosion tests, varying the corrosive medium and temperature, are shown in Figure 3. In the as-received state, the sample has an average hardness value of around 267.5 ± 11.7 kgf/mm^2^, which agrees with the literature [2,4,28].

It is possible to observe that the Vickers hardness values were not altered with the performed tests. Small changes are not significant and are within the considered confidence interval. The results agree with the literature [2,20], since the passive film does not alter the “bulk” hardness of the material.

**Figure 4:**
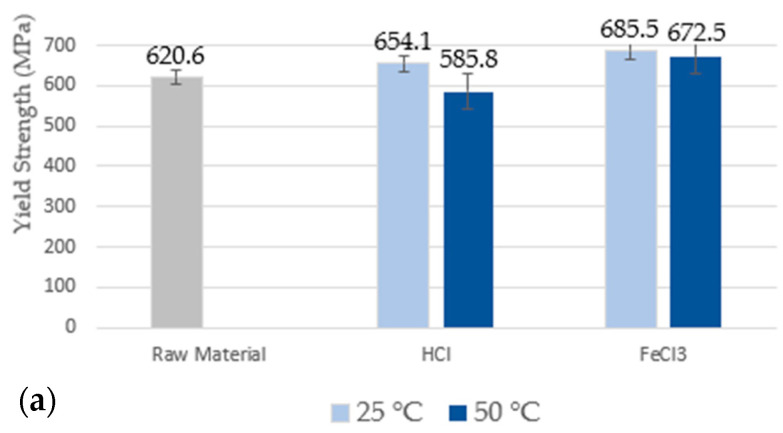

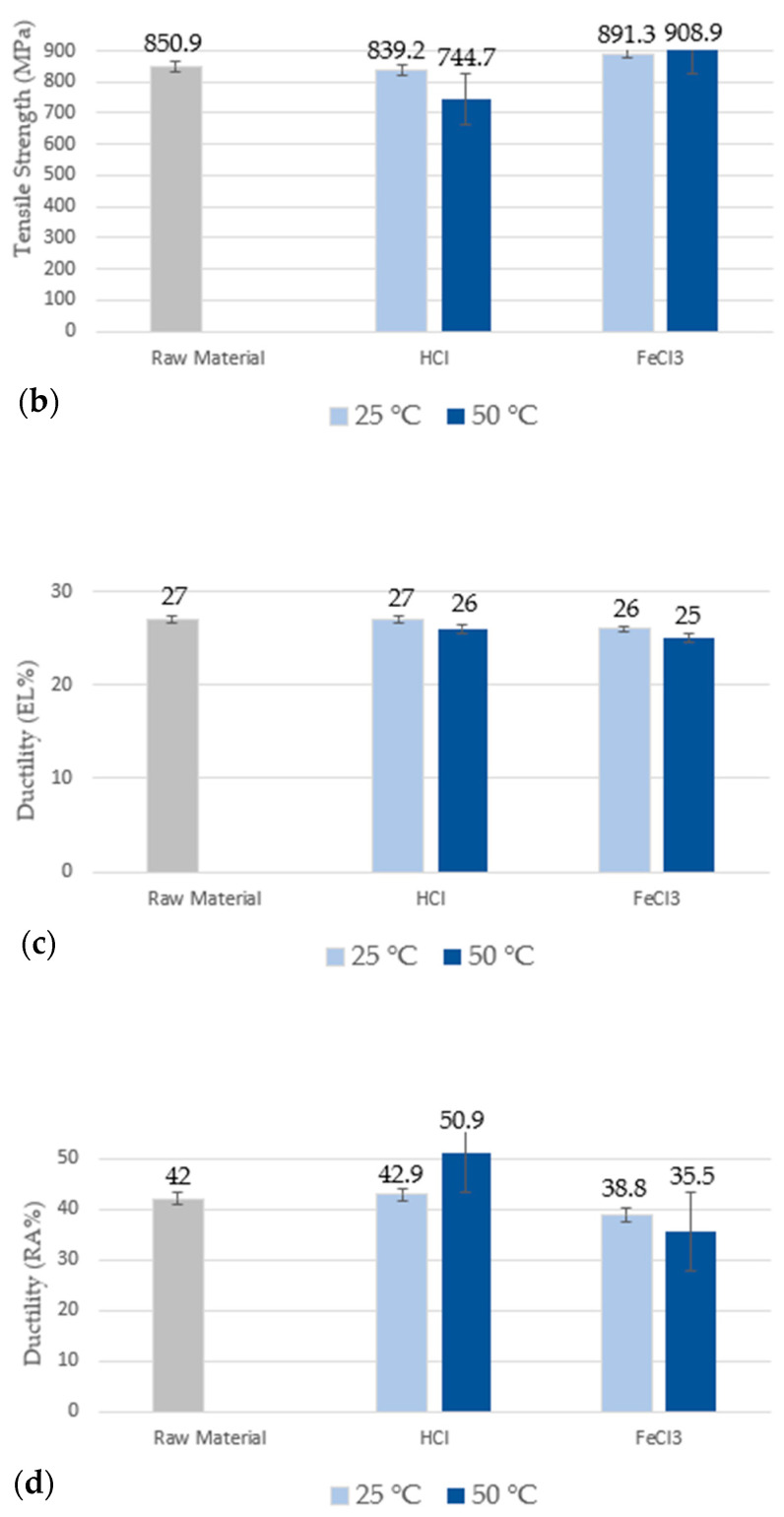
Values of yield strength (**a**), tensile strength limit (**b**), EL% ductility (**c**), and RA% ductility (**d**) obtained from the tensile test.

The results of the yield strength, tensile strength, and ductility of duplex stainless steel S31803, before and after the corrosive process, are shown in Figure 4. It is observed that at RT, in both corrosive media, the yield strength flow has a slight tendency to increase. However, with increasing temperature, there is a tendency to decrease, as in Figure 4a. These changes were small and were not able to make the material brittle below the specified limit of 450 MPa, as in the literature [2,4,29].

For the average tensile strength limit values, as in Figure 4b, it is possible to observe that for samples subjected to the HCl medium, the values show a tendency to decrease with the increase in temperature, while for the samples subjected to the FeCl_3_ solution, the values tend to increase. However, these changes are not significant and are within the determined confidence interval. The corrosive process was not able to cause any type of fragility in the material, since all values remained within the range specified in the literature, i.e., 620 MPa [2,4,29].

Figure 4c,d shows the ductility (%EL) and ductility (%RA) data obtained from the sample as received and after being subjected to corrosive media. Tests performed at RT show slightly higher data values when compared to tests performed at a temperature of 50 °C. The increase in temperature can promote greater deterioration of the passive layer, accelerating the corrosive process and a possible decrease in the values of mechanical properties [2]. According to the literature [2,4,29] the ductility values (EL%) and (RA%) are around 25% and 35%, respectively, which corroborates the results obtained.

### 3.4. Uniform Corrosion Rate

The uniform corrosion rate values, determined for the samples subjected to the hydrochloric acid and ferric chloride media, are shown in Table 2. The specimens subjected to the test using HCl as the medium were classified, according to the NACE standard 0775, as severe-type corrosion at both temperatures. However, for the ferric chloride solution, a significant increase in corrosion is observed, from almost zero classification at RT, to severe in the test at 50 °C.

When metallic materials are heated, the oxide film on the surface ruptures. Up to a certain temperature, which depends on the material and medium, the oxide layer is dense, adherent and protects the metallic alloy against further attack. When this temperature is exceeded, the oxide layer breaks and detaches from the surface of the material, thus losing its protective capacity [2,11].

It is known that the ferric chloride solution is highly aggressive [19,30,31]; with increasing temperature, the corrosive process is accelerated, resulting in a decrease in the protection of the passive film.

### 3.5. Pitting Corrosion Rate

**Figure 5:**
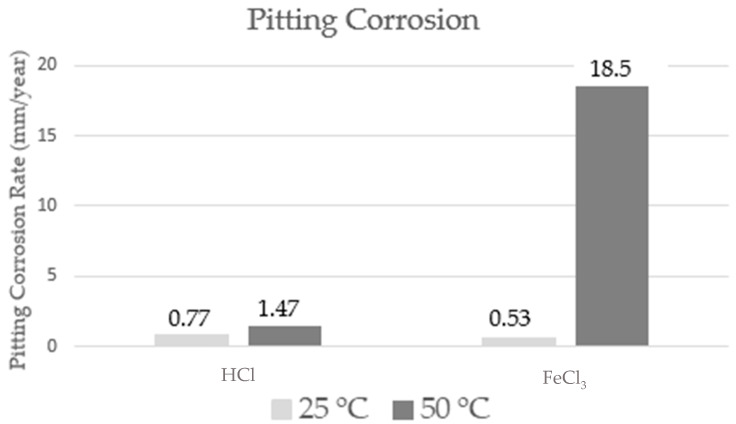
Pitting corrosion rate (mm/year) of the samples submitted to the corrosive medium of HCl and FeCl_3_, at temperatures of 25 and 50 °C. The scale on the right refers only to the FeCl_3_ medium at 50 °C.

The pitting corrosion rate values, determined for the samples subjected to hydrochloric acid and ferric chloride media, at temperatures of 25 and 50 °C, are shown in Figure 5. Regarding the qualitative analysis, all specimens presented values for the pitting corrosion rate which are classified as showing severe-type corrosion (>0.38 mm/year), according to NACE RP 0775 [18].

It can be seen from the results obtained that the increase in temperature significantly influences the pitting corrosion rate. According to the NACE RP 0775 standard [18], higher corrosion rate values are found for deeper pits. The results found are in accordance with the literature [30], since the increase in temperature accelerates the corrosive process.

**Figure 6:**
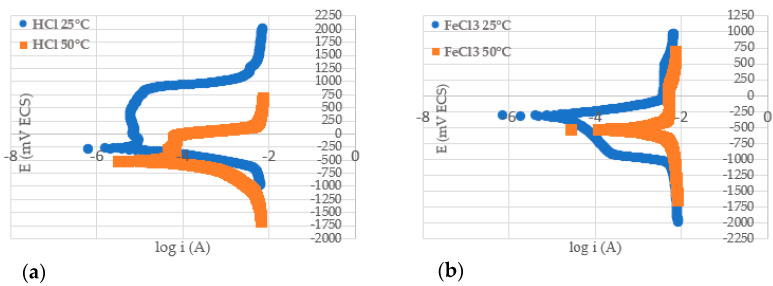
Potentiodynamic polarization curves of duplex stainless steel S31803 subjected to corrosion testing with 1.5% hydrochloric acid (**a**) and 6% ferric chloride (**b**).

A very interesting fact was observed for samples subjected to different corrosive media. For the HCl solution, the pitting corrosion rate value doubled with increasing temperature, while for the samples subjected to the FeCl_3_ solution, the values increased by 35 times. Note in Figure 6 that the scale on the right refers only to the FeCl_3_ medium at 50 °C. This fact can be explained because the increase in temperature increases the susceptibility to pitting corrosion, due to the destabilization of the passive layer. Corrosion is a thermally activated process [31].

Authors, such as Souza et al., [30] studied corrosion by chlorides in duplex stainless steel samples. They found that pitting corrosion occurs in this material up to a temperature of 60 °C, and that even at this temperature, chloride ions in aggressive media penetrate the passivating film, causing it to break through localized attacks. This passivating film mainly consists of two layers, namely an outer hydrated layer containing hydroxide ions and an inner layer containing iron and chromium oxide.

Pitting corrosion is characterized by a localized corrosive attack and starts with the breakdown of the passive film, from ions, such as chloride, which, due to their high electronegativity, attract cations from the passive film, breaking it and forming metallic cations. Once metallic cations have formed, chloride anions migrate to the inner region of the pit, in order to balance the electrical charges, where they again attack the passive layer. As the tendency towards an increase in chloride concentration leads to the hydrolysis of water, resulting from the formation of hydrochloric acid and a reduction in the local pH, this factor tends to increase the corrosion rate, leading to an increase in the concentration of chlorides and making pitting corrosion an autocatalytic process [4,20].

### 3.6. Polarization Test

**Figure 7:**
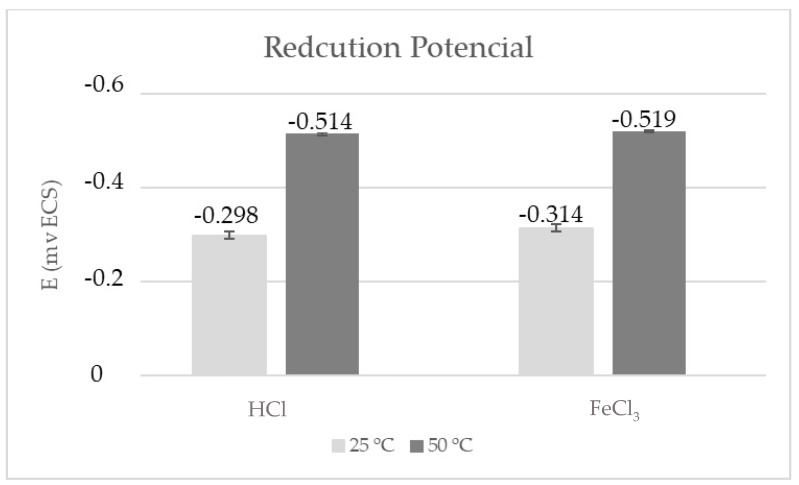
Reduction potential values obtained from the potentiodynamic polarization test.

The curves obtained from the potentiodynamic polarization test and the values of the reduction potential, determined for duplex stainless steel S31803 in hydrochloric acid and ferric chloride medium, at temperatures of 25 and 50 °C, are shown in Figure 6 and Figure 7. The potentials found for the HCl solution, at temperatures of 25 and 50 °C, were −0.289 V ± 0.006 and −0.514 V ± 0.013, respectively, while for the FeCl_3_ solution, they were −0.314 V ± 0.017 and −0.519 V ± 0.050, respectively. Colli [28] presented potential values from the potentiodynamic corrosion technique in 1M HCl solution at different temperatures. At a temperature of 30 °C, a potential around −420 mV was determined, at a temperature of 40 °C, a potential of −470 mV was determined, and at a temperature of 50 °C a potential of −490 mV was determined. These values are comparable with the results obtained.

As previously mentioned, the corrosive process accelerates with increasing temperature, since the passive film presents deformations in its formation process [30]. This behavior was observed in both of the corrosive media studied, since the increase in temperature promoted higher corrosion rates, both uniform (Table 2) and pitting (Figure 5), and a reduction in the reduction potential (Figure 7) as corroborated by the literature [30]. Therefore, the results presented in the polarization test confirm the results presented by the immersion test. For the two tests carried out, it is observed that the higher the temperature, the higher the corrosion rate.

**Figure 8:**
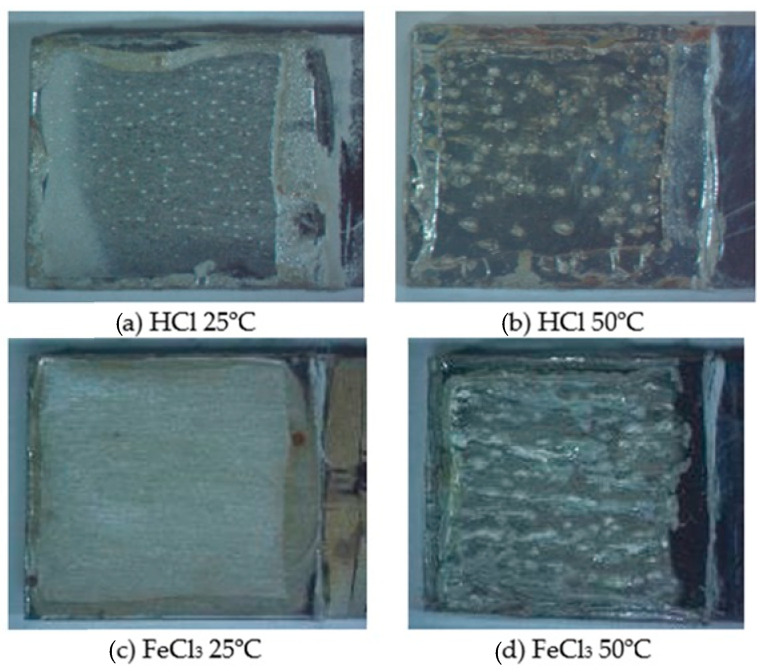
Macrograph of duplex stainless steel S31803 after the potentiodynamic polarization test in 1.5% hydrochloric acid solution (**a**,**b**) and 6% ferric chloride (**c**,**d**), at temperatures of 25 °C (**a**,**c**) and 50 °C (**b**,**d**).

The macrographs of duplex stainless steel S31803 after the potentiodynamic polarization test in 1.5% hydrochloric acid and 6% ferric chloride solutions, at both temperatures, are shown in Figure 8. In all macrographs, it is possible to identify corrosion pits, which are more evident in the samples subjected to the FeCl_3_ solution at 50 °C. This more pronounced corrosion, for the sample subjected to the ferric chloride solution at 50 °C, was also verified in the immersion corrosion test, as shown in Figure 2, Figure 5, and Figure 6. The presented macrographs, as in Figure 8, corroborate the results obtained by the immersion test and potentiodynamic polarization, in both solutions, since, with the increase in the number of pits, an increase in the uniform corrosion rate and a reduction in the potential was also observed, as shown in Table 2 and Figure 5, in addition to of a tendency to decrease in the values of the mechanical properties of the material, as in Figure 3 and Figure 4.

## 4. Conclusions

In this work, corrosion was evaluated in S31803 duplex stainless steel subjected to media containing chloride ions, due to the application of this material in this environment in the petroleum industry. The steel in the as-received condition has a microstructure composed of two phases, namely ferrite and austenite, oriented in the rolling direction and with an approximate balance of 50% in volume of fractions of the phases present. Samples subjected to the HCl corrosive medium showed a uniform and pitting corrosion rate, classified as severe, according to NACE RP0775 [17], at temperatures of 25 and 50 °C. The pitting corrosion rate determined for these temperatures was 0.77 and 1.47 mm/year, respectively. Similar behavior was observed for both corrosion rates of the samples subjected to the FeCl_3_ corrosive medium. The increase in temperature provides a significant increase, increasing the uniform corrosion rate by around 0.9 mm/year and the pitting corrosion rate by around 18 mm/year. The values of the mechanical properties show a small tendency to decrease when the corrosion tests are being carried out. The dissolution potentials decrease with the increase in the test temperature, thus characterizing a greater corrosion in the samples with the increase in the temperature.

## Figures and Tables

**Table 1:** Percentage of steel phases of duplex stainless steel S31803 in the rolling and normal directions.

Lamination		Normal	
%Ferrite	%Austenite	%Ferrite	%Austenite
46.2 ± 4.3	53.8 ± 4.3	56.1 ± 1.3	43.9 ± 1.3

**Table 2:** Uniform corrosion rate (mm/year) of the samples subjected to the corrosive mediums of HCl and FeCl_3_, at temperatures of 25 and 50 °C.

Solution/Temperature	Corrosion Rate
HCl 25 °C	0.85
HCl 50 °C	0.92
FeCl_3_ 25 °C	0.0006
FeCl_3_ 50 °C	0.93

## Data Availability

Data are contained within the article.
